# Technology investigation on time series classification and prediction

**DOI:** 10.7717/peerj-cs.982

**Published:** 2022-05-18

**Authors:** Yuerong Tong, Jingyi Liu, Lina Yu, Liping Zhang, Linjun Sun, Weijun Li, Xin Ning, Jian Xu, Hong Qin, Qiang Cai

**Affiliations:** 1 Institute of Semiconductors, Chinese Academy of Sciences, Beijing, China; 2 Shenzhen DAPU Microelectronics Co., Ltd., Shenzhen, China; 3 National Engineering Laboratory for Agri-product Quality Traceability, Beijing Technology and Business University, Beijing, China

**Keywords:** Time series analysis, Classification, Prediction, Evaluation models

## Abstract

Time series appear in many scientific fields and are an important type of data. The use of time series analysis techniques is an essential means of discovering the knowledge hidden in this type of data. In recent years, many scholars have achieved fruitful results in the study of time series. A statistical analysis of 120,000 literatures published between 2017 and 2021 reveals that the topical research about time series is mostly focused on their classification and prediction. Therefore, in this study, we focus on analyzing the technical development routes of time series classification and prediction algorithms. 87 literatures with high relevance and high citation are selected for analysis, aiming to provide a more comprehensive reference base for interested researchers. For time series classification, it is divided into supervised methods, semi-supervised methods, and early classification of time series, which are key extensions of time series classification tasks. For time series prediction, from classical statistical methods, to neural network methods, and then to fuzzy modeling and transfer learning methods, the performance and applications of these different methods are discussed. We hope this article can help aid the understanding of the current development status and discover possible future research directions, such as exploring interpretability of time series analysis and online learning modeling.

## Introduction

Time series are a set of observations made and recorded at different points in time (Misra & Siddharth, 2017). It is ubiquitous in real life. Whether measured during natural processes (weather, sound waves) or artificially generated processes (stock, robots), most real-world data contain time elements (Langkvist, Karlsson & Loutfi, 2014). Moreover, time series data are being produced in different fields at an unprecedented scale and speed. Therefore, knowledge discovery from time series has considerable potential. Because of its unique sequence characteristic, time series analysis is considered one of the ten most-challenging problems in the field of data mining (Yang & Wu, 2006), becoming a prevalent research topic that has attracted the attention of many researchers over the years (Schreiber, 1999; Osmanoglu et al., 2016). In time series analysis, common data sets are often used, such as UCR time series classification archive (https://www.cs.ucr.edu/ eamonn/time_series_data_2018/), Awesome Public Dataset (https://github.com/awesomedata/awesome-public-datasets) and CEIC (https://www.ceicdata.com/zh-hans).

**Figure 1:**
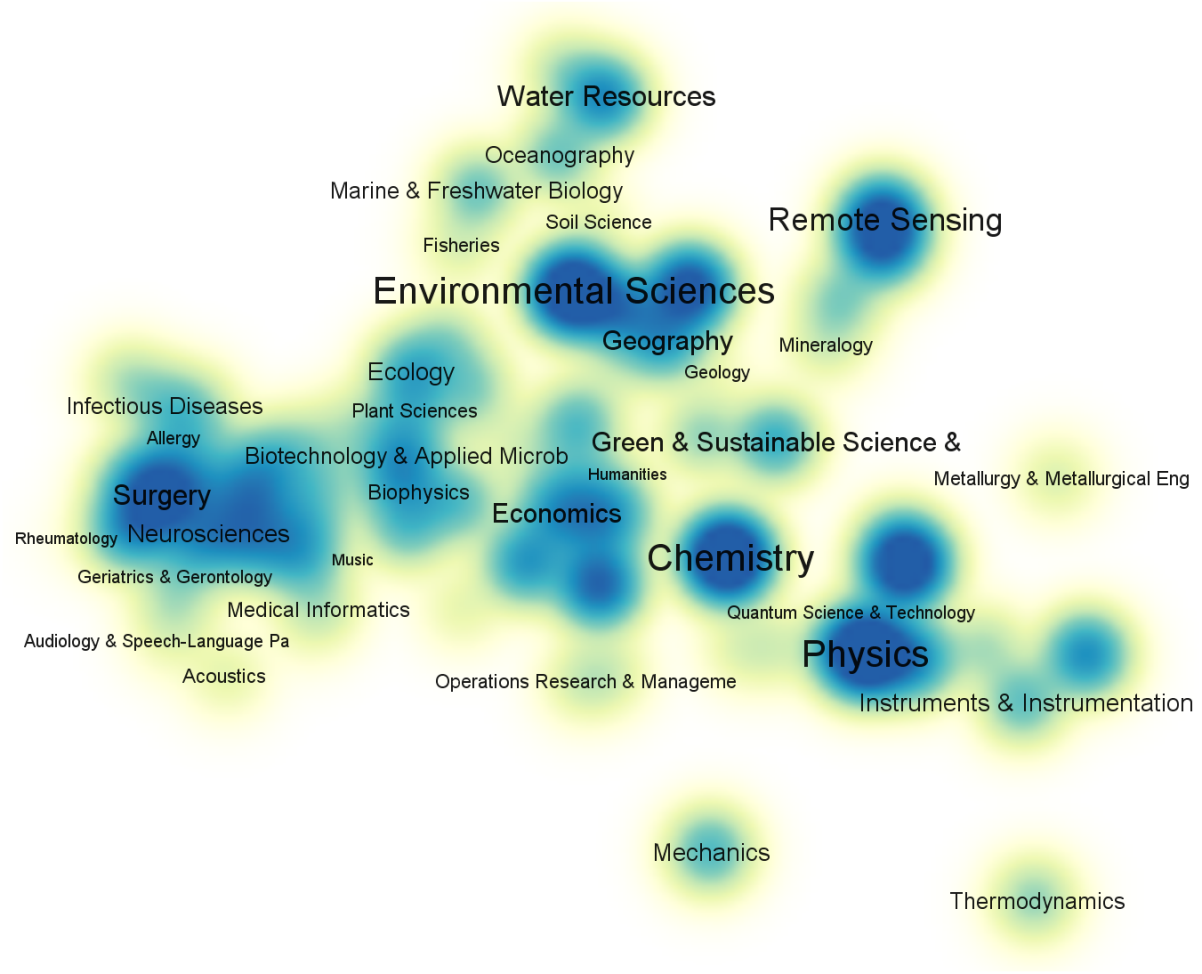
Subject category co-occurrence map of time series literatures (2017–2021).

To gain a comprehensive understanding of the current status of time series application, we use time series as a keyword to search the Web of Science Core Collection and collect 120,000 references published between 2017 and 2021. Then, we use VOSViewer (Leiden University, The Netherlands) to visualize anaysis result: the subject category co-occurrence map of first-level disciplines as shown in Fig. 1.

To gain a clearer understanding of the application fields of time series, we remove the two subjects with the highest number of matches, *i.e.,* Engineering and Computer Science, both of which have a high total link strength; this can be attributed to the fact that these two subjects are often used in research as analysis tools for other domains. The 120,000 publications contain 161 unique level-1 subjects in total. From Fig. 1, we can see that time series has an extensive range of applications.

Time series has been widely used in many fields (Jiang, 2015) such as environmental sciences (Shahane, Thomas & Bock, 1977; Gluhovsky & Agee, 2007), chemistry (Bates et al., 2014), physics (Song & Russell, 1999), mathematics (Corradi, 1995; McDonald & Alan, 1986), biomedical (Bar-Joseph, 2004; Zeger, Irizarry & Peng, 2006), meteorology (Ghil et al., 2002), astronomy (Zhu, 2017), finance (Sezer, Gudelek & Ozbayoglu, 2020), and other fields (Li et al., 2019). Specifically, in medicine, medical time series test data can be used to diagnose diseases (for example, diagnoses of heart disease Kadous & Sammut, 2005), predict disease counts (Talaei-Khoei & Wilson, 2019), evaluate the impact of interventions on public health over time (Lopez, Steven & Antonio, 2017), and analyze gene sequences to gain a deeper understanding of the human body (Bar-Joseph, 2004). Further, in environmental science and radiology, researchers can use observational data to analyze hydrometeorology (Shahane, Thomas & Bock, 1977), climate change (Gluhovsky & Agee, 2007), rainfall prediction (Barrera-Animas et al., 2022), X-rays, and gamma rays (Protheroe & Hocking, 1988). Finally, financial and traffic data are commonly used to predict market fluctuations (Idrees, Alam & Agarwal, 2019), stock prices (Chen, Cheng & Jong Teoh, 2007; Spiro et al., 2018; Li, Wu & Wang, 2020), passenger flow (Ye et al., 2020), *etc.* Time series is a ubiquitous data type in our daily lives, and the analysis thereof holds great value.

Time series applications are present in every aspect of our lives, computational statistics and data analysis will give us a new perspective and help us gain a deeper understanding of the world.

### Motivation

Time series is an important data object, used in an extensive range of research, including classification, prediction, clustering, similarity retrieval, anomaly detection, and noise elimination (Kalpakis, Gada & Puttagunta, 2001). The analysis and investigation of its current research applications can provide a comprehensive research review to aid future researchers in understanding the current development state of time series-related algorithms.

**Figure 2:**
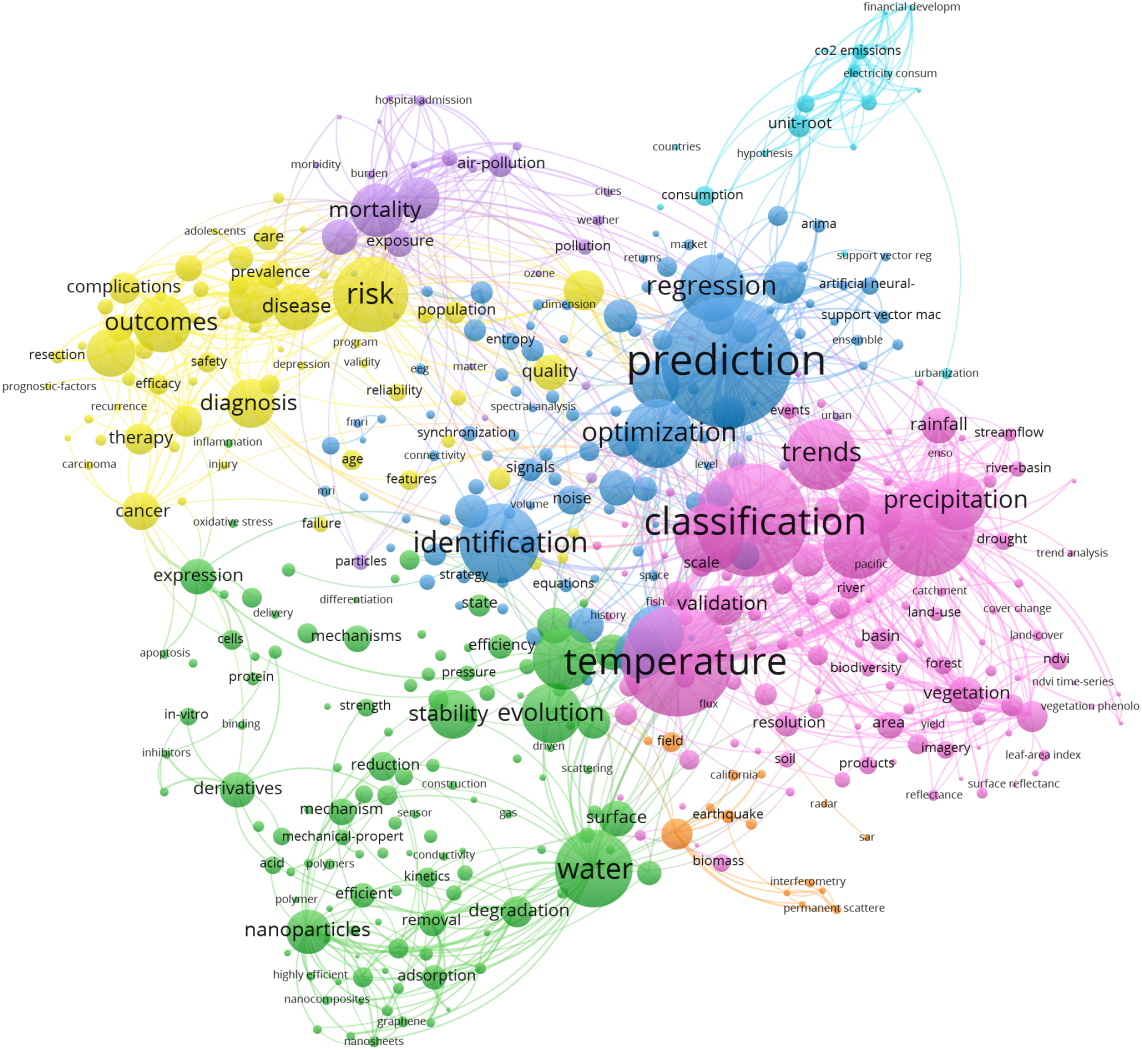
Keyword co-occurrence map of time series literatures (2017–2021).

To identify the trending topics in current time series research, we further analyze the chosen studies. After removing generic terms like time series, time, analysis of time series, *etc.*, we obtain a co-occurrence map by using literature keyword, shown in Fig. 2.

The font size in the figure is related to the frequency of occurrence of keywords. The larger the font, the higher the frequency of occurrence. There are approximately seven clusters in the figure, representing algorithms and different application domains. Two main research topics are identified, namely, classification and prediction. Because this article focuses on the analysis of time series algorithms, we will present our analysis and conclusions based on the technical development route of classification and prediction algorithms and discuss relevant areas for subsequent research.

### Main contribution

The main contributions of this article can be summarized as follows:

• a comprehensive analysis of prevalent topics in the field of time series;
• an investigation into the progress of time series classification and prediction problems in recent years, highlighting several technical development routes that are widely studied in the field, and discussing the improvement and optimization of these algorithms;
• a comparison of the performance of the different algorithms on multiple datasets, concluding with their advantages and disadvantages;
• and finally, an analysis of the challenges and future development tendencies of time series classification and prediction problems.

### Methods and materials

The following is the process of our study. First, a literature analysis tool is used to identify current popular research topics. We use VOSViewer to analyze the time series literature through keywords to examine the areas of greatest interest. These topics are classified into 478 categories, and the two research directions with the highest frequency are “classification” and “prediction”. Then, the relevant scientific literatures for the identified categories are located. We review related papers on time series classification and prediction and select 87 literatures with high relevance and high citation for analysis. The scientific databases used in the search include Web of Science Core Collection, IEEE Xplore, ACM Digital Library, Springer Link, and ScienceDirect. Finally, according to the literatures, important technical development routes are extracted, and detailed analysis and summary are carried out.

### Structure of this survey

The remainder of this article is organized as follows. ‘Related Work’ provides an introduction of related work of time series investigation and the comparison of our survey with other traditional surveys and review articles. ‘Preliminaries’ describes the fundamentals of time series classification and prediction tasks. ‘Time Series Classification’ elaborates on the development route of time series classification and prediction algorithms, by comparing their performances, analyzing the challenges being faced, and discussing future development trends. Finally, the ‘Conclusion’ concludes the article.

## Related work

Knowledge discovery in time series is an important direction for dynamic data analysis and processing. The urgent need to predict future data trends based on historical information has attracted widespread attention in many research fields. In the past few decades, many studies have summarized time series research methods from different perspectives. Table 1 summarizes the existing time series surveys and their contributions.

**Table 1:** Related time series surveys.

Theme	Related surveys	Topic	Key contributions
Prediction	(De Gooijer & Hyndman, 2006)	prediction	Review the time series prediction research of the past 25 years.
	(Mehrmolaei & Keyvanpourr, 2015)	time series mining; event prediction	Classify and evaluate event prediction methods in time series.
	(Nyein Naing & Htikemuhammad Yusof, 2015)	time series prediction; machine learning	Review the time series prediction of machine learning technology in different states spanning ten years.
	(Mahalakshmi, Sridevi & Rajaram, 2016)	prediction; time series mining	Provide a detailed survey of various techniques used to predict different types of time series datasets, and discuss various performance evaluation parameters used to evaluate predictive models.
	(Deb et al., 2017)	prediction; machine learning; energy prediction	A comprehensive review of existing machine learning techniques used to predict time series energy consumption.
	(Tealab, 2018)	prediction; nonlinear time series; neural network	Summarize the research progress of artificial neural network methods in time series prediction models.
	(Bose & Mali, 2019)	prediction; fuzzy time series	Summarize and review the contributions in the field of fuzzy time series prediction in the past 25 years.
	(Hajirahimi & Khashei, 2019)	prediction; mixed structure	Analyze various hybrid structures used in time series modeling and prediction.
	(Salles et al., 2019)	prediction model; non-stationarity; conversion method	Review and analyze the conversion methods of non-stationary time series, and discuss their advantages and limitations on time series prediction problems.
	(Sezer, Gudelek & Ozbayoglu, 2020)	prediction; deep learning; finance	Provide research on deep learning in the field of financial time series prediction.
	(Lim & Zohren, 2021)	counterfactual prediction; deep neural networks	Survey encoder–decoder designs for time series forecasting and recent developments in hybrid deep learning models.
	(Liu et al., 2021)	Intelligent predictors; Hybrid modeling strategies	Analyze various components and combinations in mixed models for time series forecasting.
Classification	(Radha & Divya, 2017)	classification; data mining technology	Research multiple time series and classification techniques and investigate various data mining methods for disease prediction.
	(Fawaz et al., 2019)	deep learning; time series classification	Conduct empirical research on the latest deep neural network architecture for time series classification, and analyze the latest performance of deep learning algorithms for time series classification.
	(Abanda, Mori & Lozano, 2019)	classification; distance	Summarize the development of distance-based time series classification methods.
	(Ali et al., 2019)	clustering; classification; visualization; visual analysis	Clarify the main concepts of using clustering or classification algorithms in the visual analysis of time series data.
Data mining	(Chung Fu, 2011)	data mining; representation; similarity; segmentation; visualization	Comprehensively review the existing research on time series data mining and divide it into research directions such as representation and indexing, similarity measurement, segmentation, visualization, and mining.
	(Fakhrazari & Vakilzadian, 2017)	data mining; machine learning	Summarize the existing data mining techniques for time series modeling and analysis and divide the main research directions of time series into three sub-fields: dimensionality reduction (time series representation), similarity measurement, and data mining tasks.
Clustering	(Rani & Sikka, 2012)	clustering; data mining; dimensionality reduction; distance measurement	Investigate the clustering of time series in various application fields such as science, engineering, business, finance, economics, health care, and government.
	(Seyedjamal, Saeed & Wah, 2014)	time series clustering; subsequence	Review the definition and background of subsequence time series clustering.
	(Aghabozorgi, Seyed Shirkhorshidi & Ying Wah, 2015)	clustering; distance measurement; evaluation measures	Reveal the four main components of time series clustering, investigating the improvement trends in the efficiency, quality, and complexity of clustering time series methods over the past decade.
	(Teichgraeber & Brandt, 2022)	clustering; representative periods	Summarize time series analysis methods used in energy system optimization models.
Similarity measure	(Chen, Liu & Sun, 2017)	time series data mining; time series similarity; mining accuracy	Analyze the advantages and disadvantages of current time series similarity measures, and the application of similarity measures in the clustering, classification, and regression of time series data.
	(Zheng-Xin et al., 2017)	multivariate time series; data mining; similarity; similarity search	Summarize the existing time series similarity measures, compares different methods of multivariate time series similarity searches, and analyze their advantages and disadvantages.
Deep learning	(Langkvist, Karlsson & Loutfi,, 2014)	unsupervised feature learning; deep learning	Review the latest developments in deep learning and unsupervised feature learning for time series problems.
	(Xu-Dong, 2019)	deep learning; prediction; classification; anomaly detection	Summarizes the latest deep learning methods for time series prediction, classification, and anomaly detection from the aspects of application, network architecture, and ideas.
	(Lara-Benítez, Carranza-García & Riquelme, 2021)	deep learning; forecasting	Evaluate the performance of several deep learning architectures on multiple datasets.
Change detection	(Zhu, 2017)	time series change detection	A comprehensive review of the four important aspects of the Landsat time series-based change detection research, including frequency, preprocessing, algorithm, and application.
	(Namoano et al., 2019)	online change detection; anomaly detection; time series segmentation	Summarize the main techniques of time series change-point detection, focusing on online methods.
Others	(Patton, 2012)	correlation; reasoning; multivariate model; semi parametric estimation	Investigates the estimation, inference methods, and goodness- of-fit test based on copula-based economic and financial time series models, as well as the empirical application of copula in economic and financial time series.
	(Sang, 2013)	hydrological time series analysis; wavelet transform	Summarizes and reviews the research and application of wavelet transform method in hydrological time series from six aspects.
	(Nordman & Lahiri, 2014)	experience likelihood	Summarize the progress of the experience likelihood of time series data.
	(Tang et al., 2015)	complexity test	Discuss the complexity testing technology of time series data.
	(Scotto, Wei & Gouveia, 2015)	autocorrelation function (ACF); count; sparse operator	Investigate the development of the field of integer-valued time series modeling, and review the literature on the most relevant sparse operators proposed in the analysis of univariate and multivariate integer-valued time series with limited or unlimited support.
	(Maçaira et al., 2018)	regression analysis; artificial intelligence; exogenous variables; prediction scheme	A systematic literature review of time series models with explanatory variables.
	(Papo, 2021)	irreversibility; time-reversal symmetry	Review and compare important algorithms for testing the irreversibility of time series.

In contrast to the above works, we focus on the development direction of time series technical routes, try to track the most primitive methods of each technical route, study the improvement ideas and improvement strategies of subsequent methods, and compare the advantages and disadvantages of various technical routes and methods. Finally, we provide new ideas for future work.

## Preliminaries

### Categories of time series

Using data characteristics, time series can be classified into five categories:

1. *Variables*: According to the number of variables, time series can be divided into univariate and multivariate time series. Univariate time series only contains a single variable, while multivariate time series contains multiple variables. For example, Kadous & Sammut (2005) use ECG (electrocardiogram) to predict whether patients suffer from heart disease; here ECG can be regarded as a univariate time series. Knape et al. (Lopez, Steven & Antonio, 2017) use weather and climate data to analyze its influences on the animal population. These two variables form a multivariate time series.
2. *Continuity*: Time series can be classified as discrete or continuous time series. For example, a gene sequence can be regarded as discrete time series (Göb, 2006), while hourly power demand is a continuous time series.
3. *Stability*: Based on the stability of a time sequence, time series can be categorized as a stationary time series or non-stationary time series. The statistical law states that a stationary time series will not change over time. Its sequence diagram intuitively shows random fluctuations around a constant value, within a bounded fluctuation range, with no obvious trends or periodic characteristics. The common periodic function is a typical stationary time series. However, in real life, non-stationary time series constitute the majority, with examples like wind intensity.
4. *Distribution*: Based on the sequence distribution, a time series can be divided into Gaussian and non-Gaussian time series.
5. *Chaos*: The generation of a chaotic time series is related to its initial conditions, where a change in the initial state of the system may lead to a critical state or inflection point of the interconnected system, significantly impacting on the performance of interconnected system. For example, the action of opening a window or door will affect the power consumption of an air conditioning system (Kim, 2017).

### Related definitions

To explain time series and its methods in clearer manner, some definitions involving time series are introduced below.

**Definition 1**. *Univariate time series*: A univariate time series, *s* = *t*_1_, *t*_2_, …, *t*_*L*_, is an ordered set of length *L*.

**Definition 2**. *Multivariate time series*: A multivariate time series, *X* = (*x*_1_, *x*_2_, …, *x*_*T*_), is a sequence vector, where each element *x*_*i*_ is a univariate time series, with differing lengths, *X* has *T* variables, with the ith variable being *x*_*i*_.

**Definition 3**. *Subsequence*: Given a time sequence *s* with length *L*, *s*_*sub*_ = *s*[*m*, *m* + *n* − 1] is a subsequence with a length *n* < *L*. The starting point of the subsequence is the position *m* in *s*, and the position *m* + *n* − 1 is the end point, represented as *s*_*sub*_ = *t*_*m*_, …, *t*_*m*+*n*−1_, where, 1 ≤ *m* ≤ *L* − *n* + 1.

**Definition 4**. *Similarity degree*: For two time series, *b* and *s*, (assuming |*b*| ≤ |*s*|), the similarity degree for them can be computed by *Sim*(*b*, *s*) = *min*{*dist*(*b*, *s*_*i*_)}, where *s*_*i*_ is an arbitrary subsequence of *s* that satisfies the condition |*b*| = |*s*_*i*_|.

**Definition 5**. *Shapelet*: A shapelet is a subsequence of time series, *s*, with the strongest discriminative ability. Specifically, the shapelet can be represented by *p* = (*b*, *δ*, *c*), where *b*, *δ*, *c* are the subsequence, threshold, and class label, respectively. If an unknown time series satisfies the condition *Sim*(*p*, *s*) ≤ *δ*, then it can be categorized into class *c*.

**Definition 6**. *Euclidean distance*: Euclidean distance is a frequently used distance measurement to determine the degree of similarity of two different time series. For sequences *b* and *c*, both with length *L*, the Euclidean distance can be calculated as ${dist}_{euclidean}=\sqrt{{\mathop{\sum }\nolimits }_{i=1}^{L}({b}_{i}-{c}_{i})^{2}}$.

**Definition 7**. *Dynamic time warping (DTW)*: DTW is another widely used distance measurement method. Compared with Euclidean distance, it can compute the minimum distance between two sequences with different lengths. For its wide application, the principle will not be explained here, but the calculation is given as *dist*_*DTW*_ = *DTW*(*s*, *b*).

### Basic algorithms

In time series classification and prediction tasks, the most basic and widely used algorithms are 1NN-DTW (1 nearest neighbor dynamic time warping) and autoregressive (AR) and moving average (MA) models.

#### 1NN-DTW

The 1NN-DTW model uses DTW as distance measurement, and the simple but effective algorithm 1NN to find the nearest training sample of the current instance and assigns the same class label to the instance as the nearest training sample. This model does not require training of parameters and has high accuracy. The following pseudocode describes the procedure of 1NN-DTW.


 
_______________________ 
Algorithm 1 1NN-DTW______________________________________________________________________ 
Require: T: labeled time series dataset, the number of samples is N 
Ensure: acc: average 1NN classification accuracy 
 1:  Num = 0 
 2:  for each instance si of T do 
 3:       distance = DTW(si,T − si); 
 4:       assign the closest instance label ypred of T to s 
i; 
 5:       if ypred == ysi 
    then 
 6:           Num = Num + 1; 
 7:       end if 
 8:  end for 
 9:  acc = Num 
  N  ;___________________________________________________________________________________________    


#### AR and MA

• AR model

The model is represented as ${X}_{t}={\mathop{\sum }\nolimits }_{j=1}^{p}{a}_{j}{X}_{t-j}+{}_{t}$ and is called the p-order AR model, denoted as *AR*(*p*), where a time series value can be expressed as a linear function of its previous value, *X*_*t*_, and an impact value, *ɛ*_*t*_. This model is a dynamic model that is different from the static multiple regression model.

• MA model

The model is represented as ${X}_{t}={}_{t}+{\mathop{\sum }\nolimits }_{j=1}^{q}{b}_{j}{}_{t-j}$ and is called the q-order MA model, denoted as *MA*(*q*). The time series value, *X*_*t*_, is the linear combination of the present and past error or shock value, *ɛ*_*t*_.

## Time series classification

Unlike traditional classification tasks, the order of the time series variable is related to the input object, which makes time series classification a more challenging problem. Based on data label availability, current time series classification research mainly focuses on supervised and semi-supervised learning. Usually, supervised learning methods with labeled information show better performances. However, in real life there is a tremendous amount of unlabeled data. Therefore, some semi-supervised methods have been proposed to address this situation by constructing models using limited labeled data and a large amount of unlabeled data. In addition, some specific application scenarios have new requirements for time series classification tasks, for example, the early diagnosis of a disease, which results in a better prognosis. Early classification is used in these situations, and its goal is to classify data as soon as possible with a certain accuracy rate. This is an important extension of time series classification. This section introduces the development route of time series classification technology, analyzes the current difficulties and challenges, and mentions some expected future trends.

### Technology developments

**Figure 3:**
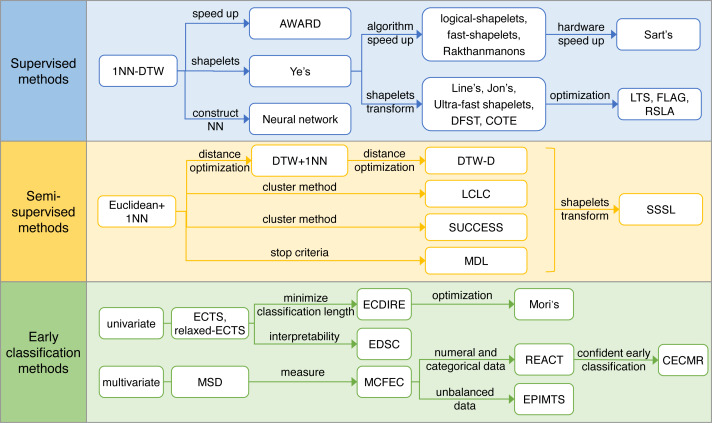
Technology development routes.

Based on the literature reviewed, we discover three development routes: supervised time series classification, semi-supervised time series classification, and early classification, which is a critical extension of the time series classification task. Fig. 3 lists the algorithms of different technology development routes.

#### Supervised learning

In early time series classification methods, the work mainly focus on the distance-based algorithm (Ding et al., 2008). The most prominent one being 1NN-DTW, which has demonstrated excellent performance in multiple tasks and datasets (Ding et al., 2008), and was once considered as an insurmountable method in time series classification (Xi et al., 2006; Ye & Keogh, 2009; Rakthanmanon & Keogh, 2013). With the deepening of related research, some algorithms with better performance, such as Rocket (Dempster, Petitjean & Webb, 2019), have achieved better results than 1NNDTW on multiple data sets. Even so, 1NNDTW is worthy of analysis and academic attention. The 1NN-DTW uses 1NN as a classifier, DTW as distance measurement criteria, and assigns the nearest training instance class label to a testing instance. This algorithm is simple, and has a high accuracy. In practice, training on the optimal hyperparameter settings, such as warping windows, is required to obtain better performance (Dau et al., 2018). However, during the classification stage, the class label of every testing instance needs to be computed by working through the entire training dataset, affording it high time complexity. The optimization of 1NN-DTW mainly concentrated on reducing classification time, by using one of three methods.

• ***Speed up***

The idea of this type of algorithm is that the effectiveness can be improved by reducing the dataset size and accelerating the computation of DTW. Through numerosity reduction and dynamic adjustment of the DTW warping window size (Xi et al., 2006), 1NN-DTW can be sped up while guaranteeing accuracy.

• ***Shapelets***

Geurts (2001) propose that a time series can be represented by its local pattern. Based on this idea, Ye & Keogh (2009) formally propose the concept of shapelets. The most important idea of shapelets is to extract the most discriminative subsequence from the whole sequence, and then making a classification by constructing a decision tree.

The advantages of the shapelet-based method are that it has strong interpretability, robustness, and low classification time complexity. Although it can be accelerated through early abandon and entropy pruning methods, the search space and time complexity of shapelets are still not negligible. Therefore, some acceleration strategies such as precomputing of reusable distance and allowable pruning (Mueen, Keogh & Young, 2011), discrete representation of subsequence (Rakthanmanon & Keogh, 2013), early abandoning Z-normalization, reordering early abandoning, reversing the query/data role, and cascading lower bounds (Rakthanmanon et al., 2012), are applied in the search of shapelets. In addition, some studies use shapelet transform to construct a new dataset from the original dataset, expecting reduced training time while retaining model interpretability and further improving accuracy (Lines et al., 2012; Hills et al., 2014). Shapelet transform separates the search procedure of shapelets and classifier construction (using the distance between shapelets and the original sequence as a new dataset), and this makes the selection of the classifier flexible.

Since the advent of shapelet transform, subsequent research has shifted focus to identify more effective ways of finding shapelets (Wistuba, Grabocka & Schmidt-Thieme, 2015; Baldán & Bentez, 2018). In contrast to constantly searching for shapelets in existing sequences, some algorithms (Grabocka et al., 2014; Bagnall et al., 2015; Hou, Kwok & Zurada, 2016; Zhao, Pan & Tao, 2020) believe that shapelets can be learned, and this changes the shapelet searching process into a mathematical optimization task, which can improve the performance of the model. However, some methods consider the performance of acceleration technology to be close to the upper bound, so other solutions must be considered, such as using multiple GPUs and FPGAs to accelerate the DTW subsequence search process (Sart et al., 2010).

• ***Constuct of a neural network***

This type of algorithm is a feature-based method, and its main idea is to train the classifier in advance. Iwana, Frinken & Uchida (2020) embed DTW into a neural network as a kernel function. In this way, the neural network can solve the problem of time series sequence recognition, such as time distortion and variable pattern length, in feedforward architecture. There have been many studies devoted to applying deep learning models to time series classification (Zheng et al., 2014), and Fawaz et al. (2019) provide a detailed introduction and summary.

Using the results from previous studies, we compare the accuracy of various methods (as shown in Table 2) with multiple public datasets which are widely used in this field (Ding et al., 2008; Rakthanmanon & Keogh, 2013; Lines et al., 2012). The performance of the shapelets learning method (LTS, FLAG, RSLA) is superior. According to the different principles used in the methods, we divide the algorithms into five categories: 1NN-DTW, shapelets, shapelets transform, shapelets learning, and neural network. In addition, the advantages and disadvantages of 1NN-DTW, shapelets, shapelets transform, and shapelets learning are compared in Table 3.

**Table 2:** Comparison of the accuracy of supervised time series classification.

Category	1NN-DTW	Shapelets	Shapelets transform		Shapelets learning			
	1NN-DTW (Ding et al., 2008)	Fast shapelets (Rakthanmanon & Keogh, 2013)	Shapelet transform (Lines et al., 2012)	COTE (Bagnall et al., 2015)	LTS (Grabocka et al., 2014)	FLAG (Hou, Kwok & Zurada, 2016)	RSLA-LS (Zhao, Pan & Tao, 2020)	RSLA-LZ (Zhao, Pan & Tao, 2020)
Adiac	60.0	54.9	29.2	76.9	49.7	74.2	**75.4**	73.9
Beef	63.3	56.7	50.0	80.0	83.3	80.0	83.3	**86.7**
Chlorine	64.8	59.1	58.8	68.6	59.4	78.0	75.0	**81.4**
Coffee	**100.0**	96.4	96.4	**100.0**	**100.0**	**100.0**	**100.0**	**100.0**
Diatom	96.4	87.9	72.2	89.2	96.7	96.4	96.7	**97.7**
DP_Little	50.3	57.8	65.4	–	**71.7**	65.7	69.1	69.8
DP_Middle	54.1	59.2	70.5	–	73.5	72.9	72.6	**73.8**
DP_Thumb	53.0	59.1	58.1	65.4	**75.7**	72.4	70.7	75.0
ECGFiveDays	78.7	99.5	77.5	99.9	**100.0**	92.0	**100.0**	**100.0**
FaceFour	82.9	92.0	84.1	71.6	95.4	90.9	92.0	**95.5**
Gun_Point	94.0	94.0	89.3	93.3	**100.0**	96.7	96.7	99.3
ItalyPower	95.2	90.5	89.2	96.2	95.9	94.6	96.5	**96.8**
Lighting7	73.9	63.0	49.3	61.6	78.1	76.7	75.3	**79.5**
Medicallmages	**74.3**	60.5	48.8	67.1	67.8	72.4	71.4	73.4
MoteStrain	86.8	79.8	82.5	84.0	85.1	88.8	**89.5**	**89.5**
MP_Little	55.2	62.1	66.4	–	**73.9**	71.8	73.6	73.6
MP_Middle	55.2	61.7	71.0	–	77.3	76.6	74.7	**78.3**
Otoliths	59.3	60.9	–	60.9	67.2	64.1	**73.4**	71.9
PP_Little	55.2	48.7	59.6	–	**72.7**	68.5	71.6	70.5
PP_Middle	50.0	56.8	61.4	–	74.9	74.0	72.7	**75.2**
PP_Thumb	51.2	58.9	60.8	–	70.1	68.4	69.8	**70.7**
Sony	73.2	68.5	–	87.7	85.3	92.8	93.2	**95.3**
Symbols	94.1	93.6	78.0	**94.7**	93.9	87.5	91.3	92.3
SyntheticC	99.3	93.6	94.3	81.0	**99.7**	**99.7**	**99.7**	99.0
Trace	**100.0**	**100.0**	98.0	**100.0**	**100.0**	99.0	98.0	**100.0**
TwoLeadECG	89.3	94.6	85.0	91.6	**99.7**	99.0	99.3	99.3

**Notes.**

A dash (-) indicates that there is no data available. The bold values represent the highest accuracy for each category.

**Table 3:** Comparison of supervised time series classification.

Category	Methods	Advantages	Disadvantages
1NN-DTW	1NN-DTW (Ding et al., 2008) AWARD (Xi et al., 2006)	Simple, no training needed	High time complexity of classification
Shapelets	Ye’s (Ye & Keogh, 2009), logical-shapelets (Mueen, Keogh & Young, 2011), fast-shapelets (Rakthanmanon & Keogh, 2013), Rakthanmanon’s (Rakthanmanon et al., 2012), Sart’s (Sart et al., 2010)	High interpretability and robustness, low classification time complexity	High time complexity of shapelets searching procedure, and for large length sequences, the time cost becomes unacceptable
Shapelet transform	Line’s (Lines et al., 2012), Jon’s (Hills et al., 2014), Ultra-fast shapelets (Wistuba, Grabocka & Schmidt-Thieme, 2015), DFST (Baldán & Bentez, 2018) COTE (Bagnall et al., 2015)	High accuracy and flexible	Long shapelets search time
Shapelet learning	LTS (Grabocka et al., 2014), FLAG (Hou, Kwok & Zurada, 2016), RSLA (Zhao, Pan & Tao, 2020)	High robustness, interpretability, discriminativeness	Long training time

1NN-DTW is the simplest, high performing method that needs no training and can correctly classify samples. However, its biggest problem is long classification times, especially for large training datasets, which makes it unsuitable for certain applications. The shapelets-based method reduces the sequence length, and thus, has a faster classification time, and achieves high interpretability and robustness. However, shapelets are discriminative features that require significant effort to find, and for large sequence lengths, the search space increases drastically. The shapelet transform method makes the choice of classifier more flexible, but it still retains the long search time problem. The shapelet learning method learns the shapelets instead of searching for them through training data, so the learned shapelets have higher robustness compared to the searched one. The disadvantage of this type of method is the long training time required.

#### Semi-supervised learning

Semi-supervised learning methods construct classifiers using a small amount of labeled data and a large amount of unlabeled data. One of the most frequently used methods is self-learning: it utilizes a small amount of labeled data to assign class labels to a large unlabeled dataset.

Wei & Keogh (2006) propose extending training data by 1NN, if the distance between the labeled data and unlabeled data is close enough, then add the unlabeled data into the training set. This is a simple and basic semi-supervised learning approach for time series classification. Based on this, the subsequent advancements can be divided into three categories.

• ***Distance***

Wei & Keogh (2006) use Euclidean distance as a similarity measurement; because DTW is a more effective distance in time series classification, it can be used to improve model performance (Chen et al., 2013). However, the ratio of DTW and Euclidean distance is proposed to be the proper distance measurement, making the algorithm more suitable for smaller data sizes and diverse negative samples. This is based on two assumptions: first, negative samples are diverse, and the negative samples may have a closer distance with positive samples; second, compared with Euclidean distance, DTW makes the inter-distance of positive samples closer.

• ***Label approach***

Other than optimizing the distance function, changing the method of adding testing data into the training dataset can also improve classification results. One possible way is to cluster negative samples. Because a robust classifier needs to be constructed using limited, labeled, positive data, partitioning the unlabeled dataset into smaller local clusters, and identifying the local clusters’ common principal features for classification can make the algorithm more reliable and productive (Nguyen, Li & Ng, 2011). Hierarchical clustering is also an effective cluster method (Marussy & Buza, 2013); first, it clusters all sequences into smaller clusters, and then uses seeds to assign labels to unlabeled data.

• ***Stopping criterion***

If a stopping criterion is too conservative (or too liberal), it is doomed to produce many false negatives (or false positives) (Begum et al., 2013). Therefore, it is important to propose a proper stopping criterion to avoid adding negative samples into the positive sample set. Begum et al. (2013) propose a parameter-free algorithm for finding a stopping criterion using the minimum description length (MDL) technique. The algorithm is stopped when the MDL becomes large, improving the classification results by optimizing the stopping criterion (Rodriguez, Alonso & Bostrom, 2001).

The accuracy of different semi-supervised methods is compared in Table 4, as collected from various studies. The overall performance of the SSSL method is the best, which shows that the method of learning shapelets through optimization algorithms is still effective in semi-supervised learning. While shapelets improve accuracy, they also improve the interpretability of the algorithm, again highlighting the importance and usefulness of shapelets.

**Table 4:** Comparison of the accuracy of semi-supervised classification methods.

Datasets	Class number	Wei (Wei & Keogh, 2006)	DTW-D (Chen et al., 2013)	SUCCESS (Marussy & Buza, 2013)	Xu (Xu & Funaya, 2015)	SSSL (Wang et al., 2019)
Coffee	2	57.1	60.1	63.2	58.8	79.2
CBF	3	99.5	83.3	99.7	92.1	100.0
ECG	2	76.3	95.3	77.5	81.9	79.3
Face four	4	81.8	78.2	80.0	83.3	85.1
Gun point	2	92.5	71.1	95.5	72.9	82.4
ItalyPow.Dem	2	93.4	66.4	92.4	77.2	94.1
Lighting2	2	65.8	64.1	68.3	69.8	81.3
Linghting7	7	46.4	50.3	47.1	51.1	79.6
OSU leaf	6	46.0	70.1	53.4	64.2	83.5
Trace	4	95.0	80.1	100.0	78.8	100.0
WordsSyn	25	59.0	86.3	61.8	63.9	87.5
OliveOil	4	63.3	73.2	61.7	63.9	77.6
StarLight Curves	3	86.0	74.3	80.0	75.5	87.2

#### Early classification

The main goal of early classification is to assign class labels as early as possible while guaranteeing a certain percentage of accuracy. It has great importance in time sensitive applications, such as the diagnosis of heart disease, as early diagnosis improves prognosis. In practical applications, due to an unclear description of the issues to be solved, the early classification of time series may cause false positives in practical applications, and the cost of false positives is very high. To solve this problem, Wu, Der & Keogh (2021) propose that the definition of early classification of time series should be clearly defined first, and it is also very important to obtain real-world publicly available datasets. According to the data type, there are two technology development routes.

• ***Univariable***

Rodriguez, Alonso & Bostrom (2001) segment a time series into intervals and then describe these intervals using relative predicates and region-based predicates. It is the first literature to mention the term early classification of time series. Although it achieves early classification by using sub-information, it does not consider ways to choose the shortest prefix to provide reliable classification results. ECTS (Xing, Pei & Yu, 2009) obtains the shortest prediction length through training, and it uses the sequence prefix to classify data under the condition of guaranteed accuracy. ECTS achieves a shorter prefix, higher accuracy, and higher effectiveness by using an accelerating algorithm. Further, Mori et al. (2016) calculate the shortest classification length for each class, while Mori et al. (2018) change this task into a mathematical optimization problem, using the accuracy and earliness as mutual optimization goals.

The above methods lack interpretability, which is useful in determining the factor affecting an object. EDSC (Xing et al., 2011) introduces shapelets and proposes local-shapelets, using kernel density estimation or Chebyshev inequality to find the threshold value of each shapelet, and then selecting the best shapelet for classification.

• ***Multivariable***

MSD (Ghalwash & Obradovic, 2012) extends the EDSC algorithm to suit a multivariable situation. It uses information gain to evaluate the goodness of shapelets, adds shapelet pruning, and abandons shapelets that has no ability to correctly classify data. This method has three disadvantages: first, it handles multivariable data in a fixed window, even though, different variables could have different shapelet positions; second, it cannot process variables with different lengths; and third, there is no connection between multiple variables.

To solve these problems, He et al. (2015) propose learning a shapelet for each variable independently, and constructing a classifier that can use multiple shapelets to classify data. Moreover, it substitutes information gain with a new measurement (F-measure). This method can solve the inter-class imbalance problem (a class containing multiple small classes, or consisting of multiple concepts) to a certain degree through inter-class clustering. Lin et al. (2015) further extend the input variables of the algorithm from continuous numerical sequences to characterized discrete sequences. He et al. (2019) use downsampling technology to solve the intra-class imbalance problem, and a clustering method to deal with the inter-class imbalance problem, which further expands the applicability of the algorithm.

In contrast, He, Zhao & Xia (2020) mainly focus on the identification of multivariable class labels as early as possible and ensures the classification accuracy higher than the probability of true label. Tables 5 and 6 compare the accuracy of some univariate early classification algorithms and multivariate early classification algorithms, respectively.

**Table 5:** Comparison of univariable accuracy in early classification.

Methods	Datasets						
	Wafer	Gun Point	Two patterns	ECG	Synthetic control	OliveOil	CBF
ECTS (Xing, Pei & Yu, 2009)	99.08	86.67	86.48	89.00	89.00	90.00	85.20
RelaxedECTS (Xing, Pei & Yu, 2012)	99.08	86.67	86.35	89.00	88.30	90.00	85.20
ECDIRE (Mori et al., 2016)	97.00	87.00	87.00	91.00	96.00	40.00	89.00
EDSC (Xing et al., 2011)	99.00	94.00	80.00	85.00	89.00	60.00	84.00

**Table 6:** Comparison of multivariable accuracy in early classification.

Methods	Datasets			
	Syn1	Syn2	Wafer	ECG
Class number	2	3	2	2
Variable number	3	4	6	2
MSD (Ghalwash & Obradovic, 2012)	0.74	0.34	0.74	0.74
MCFEC-QBC (He et al., 2015)	0.99	0.77	0.9	0.77
MCFEC-Rule (He et al., 2015)	0.98	0.74	0.97	0.78
EPIMTS (He et al., 2019)	0.98	0.99	0.96	0.84

While most of the univariable classification algorithms achieve good results (above 85%), the accuracy of multivariable algorithms do not reach that high (except EPIMTS). This can be attributed to the fact that it is difficult to consider multiple variables simultaneously and extracting the interconnection between them correctly. EPIMTS uses an ensemble method to combine these two important factors into the algorithm, allowing it to achieve the best performance.

### Challenges and future trends

This section discusses the different technology development routes in time series classification. Mainly, the research covers both traditional supervised learning methods and semi-supervised learning methods. In particular, an important extension—early classification—is proposed for specific application situations.

Although the existing work has achieved good results in time classification tasks, there are still some problems. In real life, the amount of unlabeled data exceeds that of labeled data, and its sources are more abundant. Although supervised learning yields better classification results, labeling data is expensive and time consuming. In some fields such as medical and satellite data, experts are required to label the data, making the acquisition of labeled data even more difficult. Therefore, research on semi-supervised or unsupervised methods has great value. However, according to the research reviewed for this article, very few recent studies focus on semi-supervised learning methods and unsupervised learning methods for time series classification (Wei & Keogh, 2006; Chen et al., 2013; Nguyen, Li & Ng, 2011). Managing large amounts of unlabeled data for classification tasks is a tremendous challenge we face.

## Time series prediction

Although time series prediction methods have experienced a long period of development, the rapid increase in data scale has brought severe challenges to traditional time series prediction methods, and has also seriously affected the efficiency of prediction methods. Time series prediction methods have gradually developed from simple linear regression models and nonlinear regression models based on traditional statistics to machine learning methods represented by neural networks and support vector machines. At the same time, researchers have also proposed other prediction methods for time series with different characteristics based on different theoretical foundations. Fuzzy cognitive map can deal with data uncertainty and maintain a high level of interpretability. To solve the problem of insufficient labeled data for some practical applications, transfer learning methods can be used. Two future research avenues are clear; first, dealing with rapid increase in the scale of time series data; second, choosing the most suitable model for a specific problem.

### Technology developments

**Figure 4:**
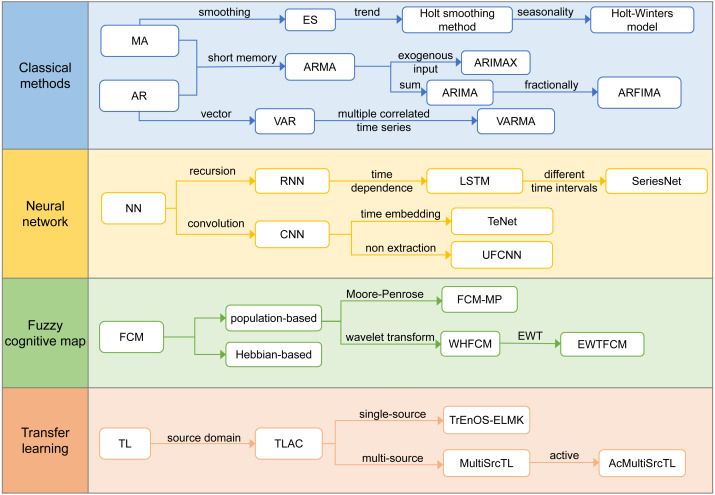
Technology development routes.

According to the reviewed literature, we have defined four technical development routes, namely, the classic algorithm, neural network, fuzzy cognitive map, and transfer learning. Figure 4 lists the development directions of the different technical routes and their resulting algorithms.

#### Classical methods

The traditional time series prediction methods are mainly used to solve the model parameters on the basis of determining the time series parameter model and using the solved model to complete the prediction work, mainly from the perspective of a stationary series, non-stationary series, or multivariate time series.

• ***Stationary series***

Russian astronomer Slutzky create and propose the moving average (MA) model (Slutzky, 1937), and British statistician G.U. Yule propose the autoregressive(AR) model (Yule, 1927) when studying sunspots. The AR model is a representation of a random process, and its output variable depends linearly on its previous value and random conditions. The purpose of the AR model is to minimize the square error between the predicted results and the actual results. Box and Jenkins propose a short memory model called autoregressive moving average (ARMA) model (Box & Jenkins, 1970). The ARMA model provide a general framework for predicting stationary observation time series data. However, it is not suitable for non-stationary time series data, and only one time series can be modeled at a time.

• ***Non-stationary series***

Non-stationary time series comprise four trends: long-term trend, cyclic trend, seasonal trend, and irregular trend. Box and Jenkins propose the autoregressive integrated moving average (ARIMA) model for non-stationary short memory data with obvious trends (Box & Jenkins, 1970). The ARIMA model has become one of the most widely used linear models in time series prediction. This model uses historical data of univariate time series to analyze its own trends and predict future cycles, but the ARIMA model cannot easily capture non-linear patterns. One or more time differentiation steps in ARIMA keep the time series data unchanged. Differentiation operations usually amplify high-frequency noise in time series data, thereby affecting the accuracy of prediction. When modeling time series with long memory dependence, a common alternative is autoregressive partial integration moving average (ARFIMA). The model is based on ARIMA and allows the difference parameters to be set to non-integer values. On the basis of the ARIMA model, the autoregressive integrated moving average (ARIMAX) model is obtained by adding exogenous input (Wangdi et al., 2010).

The exponential smoothing (ES) (Gardner, 1985) model is a time series data smoothing technique that uses past data points in a time window to smooth current data points. In contrast to the traditional MA model, the ES model uses an exponential function to assign more weight to the nearest data point, which is beneficial for processing non-stationary time series data, and is aimed at series without trend and seasonality. The Holt smoothing method (Holt, 2004; Winters, 1960), also called double exponential smoothing, is an extension of ES designed for time series with a trend but no seasonality. Chatfield (1978) propose the Holt-Winters model, which uses three smoothing steps to predict time series data. The three smoothing steps are used for level, trend, and seasonality, and are also called three exponential smoothing. The Holt-Winters model can be used for univariate time series prediction of seasonal data.

• ***Multivariate time series***

The vector autoregressive (VAR) (Mizon, 1991) model is a natural extension of the univariate AR model over dynamic multivariate time series, providing predictions superior to univariate time series models and theory-based fine simultaneous equation models. The vector autoregressive moving average (VARMA) (Athanasopoulos & Vahid, 2008) model allows several related time series to be modeled together, considering the cross-correlation and internal correlation of the series. The VARMA model fully considers the influence of each sequence on another sequence, thereby improving the prediction accuracy. This makes the predictions generated by the VARMA model more reliable for decision-making.

Traditional research methods mostly use statistical models to study the evolution of time data. For decades, linear statistical methods have dominated the prediction. Although linear models have many advantages in implementation and interpretation, they have serious limitations in capturing the nonlinear relationship in the data, which is common in many complex real-world problems.

#### Neural Network

An artificial neural network (ANN) is a flexible computing framework and general approximator that can be applied to various time series prediction problems with high accuracy. The main advantage of a neural network is its flexible nonlinear modeling ability, without the need to specify a specific model form. The popularity of ANN stems from being a generalized nonlinear prediction model. Since the advent of the simplest ANN, the ideas of recursion, nonlinear regression, and convolution continues to develop. According to the characteristics of real data, the linear and nonlinear models can be combined to construct a hybrid model to achieve better performance.

• ***Recursion***

Connor & Atlas (1991) apply a recurrent neural network (RNN) to time series prediction, using the historical information of time series to predict future results. Hochreiter & Schmidhuber (1996) proposes an improved RNN called long short-term memory (LSTM), which solves the problem of the vanishing gradient by introducing additional units that can store data indefinitely, and has shown success in single-step time series analysis. LSTM is able to address sequences of varying length and capture long-term dependencies without the same problems as traditional RNN architectures (Wilson et al., 2018). LSTM has gradually become a popular solution for learning the long-term time-dependent characteristics of original time series data, and can use a fixed-size time window to solve many time series tasks that feedforward networks cannot solve.

• ***Convolution***

Convolutional neural network (CNN) is different from RNN, which strictly uses sequential learning processes. The latter processes one data point each time to generate data representations, while the former use nonlinear filters based on multiple dataset learning representation. In each step, a filter is used to extract features from a subset of local data, so that the representation is a set of extracted features. Liu et al. (2015) use a CNN combined with time-domain embedding to predict periodic time series values; a novel model called a time-embedding enhanced convolutional neural network (TeNet), to learn the repeated occurrences in periodic time series structural elements (called abstract fragments) that have not been hidden to predict future changes. Mittelman (2015) propose a non-decimated full convolutional neural network (UFCNN) to deal with time series problems. UFCNN has no gradient disappearing and gradient explosion problems, so it is easier to train. It can be implemented more efficiently because it only involves convolution operations instead of the recursion used by RNN and LSTM.

• ***Hybrid model***

Modeling real-world time series is a particularly difficult task because they usually consist of a combination of both linear and nonlinear patterns. In view of the limitations of linear and nonlinear models, hybrid models have been proposed in some studies to improve the quality of prediction. The ARIMA model, ANN model (Peter & Zhang, 2003; Khashei & Bijari, 2010; Babu & Reddy, 2014), and multi-layer perceptron(MLP) (de O. Santos Jnior, de Oliveira & de Mattos Neto, 2019) are combined to construct a hybrid model, which has been proven by experiments to achieve better performance than a single model.

Shen et al. (2020) propose SeriesNet, using LSTM and extended random convolution to extract features with different time intervals from the time series, and combining them. This can make full use of the characteristics of the time series and help improve prediction accuracy. Compared with other models, the SeriesNet model has the best prediction accuracy in nonlinear and non-stationary datasets. In the non-stationary datasets, the error of SeriesNet decreases slowly as the size of the sliding window increases.

Table 7 compares the root-mean-square error (RMSE), the mean absolute error (MAE) and the coefficient of determination (*R*^2^) of multiple methods. We summarize the advantages and disadvantages of different methods, and the results are presented in Table 8. The hybrid model has a stronger advantage when dealing with nonlinear and non-stationary data.

**Table 7:** Performance comparison of different methods.

Methods	S&P 500 Index			Shanghai Composite Index			Hangzhou Temperature		
	RMSE	MAE	*R* ^2^	RMSE	MAE	*R* ^2^	RMSE	MAE	*R* ^2^
ANN	24.22	20.21	0.965	66.25	39.35	0.975	2.95	2.14	0.895
UFCNN (Mittelman, 2015)	24.36	19.84	0.965	93.06	57.77	0.950	**2.64**	**1.97**	**0.907**
LSTM	19.04	14.42	0.978	**63.84**	**38.05**	**0.976**	2.86	2.09	0.901
SeriesNet (Shen et al., 2020)	**17.32**	**13.15**	**0.982**	63.94	38.37	**0.976**	2.82	2.06	0.903

**Notes.**

The data are obtained from reference (Shen et al., 2020).

**Table 8:** Performance comparison of different methods.

Method category	Methods	Advantages	Disadvantages
Classical method	AR, MA, ARMA, ARIMA	Good at linear problems	Cannot handle nonlinear problems well
Traditional machine learning	SVM, LS-SVM (Suykens & Johan, 2002)	Able to solve complex time series data	Cannot handle nonlinear problems well
NN	ANN, BPNN, DE-BPNN (Wang, Zeng & Chen, 2015)	Able to handle nonlinear problems	Long-term dependence cannot be effectively preserved
LSTM	LSTM	Capable of capturing long-term dependence, structure is conducive to dealing with sequence problems	Facing the problem of gradient disappearance or gradient explosion, and it is difficult to train
CNN	CNN, UFCNN (Mittelman, 2015)	Efficient	Difficult to capture long-term dependence
Hybrid model	ARIMA-ANN (Peter & Zhang, 2003; Babu & Reddy, 2014), ARIMA-SVM (Pai & Lin, 2005; Oliveira & Ludermir, 2014), ARIMA-NN (Khashei & Bijari, 2010), ARIMA-MLP-SVR (de O. Santos Jnior, de Oliveira & de Mattos Neto, 2019), SeriesNet (Shen et al., 2020)	Better performance	High complexity

#### Fuzzy cognitive map

Fuzzy cognitive map (FCM) is a dynamic system quantitative modeling and simulation method proposed by Kosko (1986). It is a simple and powerful tool that is very useful in dynamic system simulation and analysis. FCM can be useful in time series prediction tasks that do not need to deal with exact numbers but only need approximate results (Felix et al., 2019). This method combines the characteristics of fuzzy logic and neural networks, which can effectively model the states of the system. It can simultaneously deal with the uncertainty of data and maintain a high level of interpretability. It has been demonstrated that FCM can be applied to predict time series with univariate (Lu, Yang & Liu, 2014) and multivariate (Froelich et al., 2012; Papageorgiou & Froelich, 2012a; Papageorgiou & Froelich, 2012b; Stach et al., 2005) variables.

The existing algorithms applied to train FCM belong to two main groups, population-based and Hebbian-based methods. Population-based algorithms include particle swarm optimization (PSO) (Homenda, Jastrzebska & Pedrycz, 2015; Salmeron et al., 2017), genetic algorithm(GA) (Yesil et al., 2013), memetic algorithms(Salmeron, Ruiz-Celma & Mena, 2016), artificial bee colony(ABC) (Yesil et al., 2013), and modified asexual reproduction optimization (Salmeron et al., 2019). Hebbian-based learning algorithms are seldom used for time series prediction because of their poor generalization ability.

FCM in the time series prediction domain is mostly composed of two parts, establishing the structure and learning the weight matrix. To facilitate an efficient extraction of concepts, FCM framework is constructed by using fuzzy c-means algorithm (Lu et al., 2014). When applying standard FCM to time series prediction, most of the literature (Lu, Yang & Liu, 2014; Poczeta & Yastrebov, 2014; Papageorgiou, Poczeta & Laspidou, 2015; Poczeta, Yastrebov & Papageorgiou, 2015) assumes that the weights of FCM are adjusted during the training phase and do not change with time when used for prediction. To improve the accuracy of prediction and reduce training time, some studies proposed pseudo-inverse learning and wavelet transform.

• ***Pseudo-inverse learning***

Vanhoenshoven et al. (2020) propose a new FCM learning algorithm based on the Moore Penrose inverse (FCM-MP). The unique feature of this learning method is that for the pseudo-inverse learning of the FCM weight matrix, each iteration step calculates a different set of weights. In this way, different time-varying data segments will affect the weight, and the weight will change from one iteration to the next. This algorithm improves the accuracy of prediction, does not require laborious parameter adjustments, and reduces the processing time required for training the FCM.

• ***Wavelet transform***

Although fuzzy cluster analysis has strong time series modeling capabilities, prediction methods based on fuzzy cluster analysis cannot handle non-stationary time series, and evolutionary learning methods are not suitable for large-scale time series. To overcome these two limitations, Yang & Liu (2018) propose wavelet high-order fuzzy cognitive map (WHFCM), which uses wavelet transform instead of fuzzy time series, and uses ridge regression to train. Further, empirical wavelet transform (EWT) is superior to discrete wavelet transform in time series prediction, because empirical wavelet transform is a data-driven signal decomposition algorithm. Gao, Du & Yuen (2020) propose the mixed time series prediction model based on EWT and FCM. EWT is applied to decompose the original time series into different levels to capture information of different frequencies, and to train high-order fuzzy cognitive maps to model the relationship between all generated subsequences and the original time series.

FCM has been successfully used to model and predict stationary time series. However, it is still challenging to deal with large-scale non-stationary time series with time trends and rapid changes over time. The main advantage of the FCM-based model is the human-centered knowledge representation interface. Therefore, in terms of accuracy, fuzzy admissible mapping time series modeling may not exceed the classical methods that have been studied, but FCM provides superior practical characteristics.

#### Transfer learning

Time series data usually change over time. Hence, samples collected over a long period of time are usually significantly different from each other. As such, it is generally not recommended to directly apply old data to the prediction process. For time series prediction problems, we hope to train an effective model with only a small number of fresh samples and relatively rich, old data. Therefore, to solve the problem of insufficient labeled data available in some practical applications, transfer learning methods can be used. Transfer learning is the reusing and transferring of knowledge in one field to other different but related fields. Its basic idea is to utilize the data or information of related source tasks to assist in modeling for the target task. Traditional machine learning techniques try to learn each task from scratch, while transfer learning techniques try to transfer the knowledge from some previous tasks to a target task when the latter has less high-quality training data (Pan & Yang, 2010).

Xiao, He & Wang (2012) propose a transfer learning-based analog complexing model (TLAC). First, it transfers related time series from the source domain to assist in modeling the target time series using the transfer learning technique. Ye & Dai (2018) propose a hybrid algorithm based on transfer learning, combining online sequential extreme learning machine with kernel (OS-ELMK) and integrated learning (TrEnOS-ELMK). With TrEnOS-ELMK, a single-source transfer learning algorithm is implemented. Using transfer learning, the knowledge learned from old data can be effectively used to solve the current prediction task, bridging the severe challenge brought about by long-term knowledge transfer. The distribution of time series data usually changes gradually and significantly over time; therefore, single-source transfer learning algorithm may also be confronted with the challenge of negative transfer. To solve this problem, Gu & Dai (2021) propose a new multi-source transfer learning algorithm, referred to as MultiSrcTL algorithm, and a new active multi-source transfer learning algorithm, referred to as AcMultiSrcTL algorithm.

Ye & Dai (2021) propose a deep transfer learning method (DTr-CNN) based on the CNN architecture, which inherites the advantages of CNN and tries to alleviate the problem of insufficient labeled data. This algorithm considers the similarity between the potential source dataset and the target dataset, and provides guidance for selecting the appropriate source domain. Gupta et al. (2018) propose an approach to leverage deep RNNs for small, labeled datasets *via* transfer learning.

At present, there are relatively few studies on the application of transfer learning to time series prediction. Existing research mainly focuses on the research of pattern classification. In many practical situations, the lack of labeled data may become an obstacle to time series prediction. Unlike traditional machine learning algorithms, transfer learning breaks the assumption that training data and test data must follow the same distribution. For relevant datasets with sufficiently labeled samples, the use of transfer learning framework has become a new trend, and the use of knowledge from relevant source datasets on target dataset effectively solves the problem of insufficient labeled data.

### Challenges and future trends

This section discusses the method of time series prediction. Time series data essentially reflects the changing trend of some random variables over time. The core of the time series prediction problem is to identify trends from the data, and use it to estimate the future data and predict occurrences in the next period of time. There is not one best model for all actual data, only the most suitable model from a reasonable range of models can be chosen to provide better prediction. The establishment of new time series models is still a problem that scholars will continue to study in the future, giving direction for further research in the field of time series prediction.

## Conclusion

Time series is an important data type and is generated in almost every application domain at an unprecedented speed and scale. The analysis of time series can help us understand the essence of various phenomena. We investigate current research regarding time series and find that there are few reviews for time series algorithms. In this article, we analyze the prevalent topics of time series and divide them into two categories: classification and prediction. Further, we extract the important technology development routes for time series related algorithms, and introduce every original method and its subsequent improvements. In addition, we compare the performance of different algorithms, analyze and conclude their advantages and disadvantages, as well as the problems and challenges they face.

Through our investigation, we find that the technological development has three areas: the traditional method, machine learning method, and deep learning method. In time series classification, the mainstream methods change from distance-based methods (1NN-DTW) into feature-based methods (shapelets), and finally they evolve into a mathematical optimization problem that not only improve the accuracy but also reduce the time complexity. In time series prediction, owing to the limitations of AR, MA, ARIMA, and other traditional methods that cannot cope with nonlinear problems well, neural network methods have become a popular topic, and it is expected to enhance the learning ability of models through fuzzy cognitive map and transfer learning. Despite the fact that the current research has obtained some achievements, we find some important problems during our investigation:

• For time series classification, the research on semi-supervised and unsupervised learning algorithms is insufficient. While unlabeled data is ubiquitous and available in large amounts in real life, labeling it is labor intensive and sometimes requires expert knowledge.
• For time series prediction, constructing targeted time series models to solve real-world problems is still an ongoing problem for future researchers.

In view of the current development status of time series research, we believe that there are still many possible development directions for time series analysis. For example, neural network is a very popular method for time series analysis. In most cases, its solution process is a black box, which lacks interpretability, so that the results cannot be intuitively understood, and clear and targeted optimization scheme cannot be obtained. Exploring the symbolic expression of time series with stronger interpretability is the possible development direction of time series in the future. At present, most of the time series analysis is to collect data offline for offline analysis. When the model built in the offline phase is used in the online phase, new samples are continuously obtained as the working time increases. However, most methods do not consider the use of newly obtained data, and the model cannot be updated in time. Therefore, how to update the model for real-time data is the future task of time series modeling research.

Time series has attracted much attention because of its important applications in many fields, such as disease diagnosis and traffic flow prediction. We believe that the study of time series in this article will provide a valuable reference for related research and inspire interested researchers and practitioners to invest more in this promising field.

## Additional Information and Declarations

### Data Availability

The following information was supplied regarding data availability: Our paper does not contain raw data or code.
